# Bi_2_S_3_ for Aqueous Zn Ion Battery with Enhanced Cycle Stability

**DOI:** 10.1007/s40820-019-0352-3

**Published:** 2019-12-19

**Authors:** Ting Xiong, Yinming Wang, Bosi Yin, Wen Shi, Wee Siang Vincent Lee, Junmin Xue

**Affiliations:** 1 Department of Materials Science and Engineering, National University of Singapore, Singapore, 117573 Singapore; 2 Centre for Advanced 2D Materials and Graphene Research Centre, National University of Singapore, Singapore, 117546 Singapore; 3 MIIT Key Laboratory of Critical Materials Technology for New Energy Conversion and Storage, School of Chemistry and Chemical Engineering, Harbin Institute of Technology, No. 92 West-Da Zhi Street, Harbin, 150001 People’s Republic of China

**Keywords:** Aqueous, Zn ion battery, Bi_2_S_3_, Good stability

## Abstract

Aqueous Zn ion batteries (ZIBs) are promising in energy storage due to the low cost, high safety, and material abundance. The development of metal oxides as the cathode for ZIBs is limited by the strong electrostatic forces between O^2−^ and Zn^2+^ which leads to poor cyclic stability. Herein, Bi_2_S_3_ is proposed as a promising cathode material for rechargeable aqueous ZIBs. Improved cyclic stability and fast diffusion of Zn^2+^ is observed. Also, the layered structure of Bi_2_S_3_ with the weak van der Waals interaction between layers offers paths for diffusion and occupancy of Zn^2+^. As a result, the Zn/Bi_2_S_3_ battery delivers high capacity of 161 mAh g^−1^ at 0.2 A g^−1^ and good cycling stability up to 100 cycles with ca. 100% retention. The battery also demonstrates good cyclic performance of ca. 80.3% over 2000 cycles at 1 A g^−1^. The storage mechanism in the Bi_2_S_3_ cathode is related to the reversible Zn ion intercalation/extraction reactions and the capacitive contribution. This work indicates that Bi_2_S_3_ shows great potential as the cathode of ZIBs with good performance and stability.

**Electronic supplementary material:**

The online version of this article (10.1007/s40820-019-0352-3) contains supplementary material, which is available to authorized users.

## Highlights

- Bi_2_S_3_ is proposed as a promising cathode material for rechargeable aqueous Zn ion battery.
- The Zn/Bi_2_S_3_ battery shows a reversible capacity of 161 mAh g^−1^ at 0.2 A g^−1^ and good cyclic stability of up to 100 cycles with ca. 100% retention.
- The storage mechanism in the Bi_2_S_3_ cathode is related to the reversible Zn ion intercalation/extraction reactions and the capacitive contribution.

**Electronic supplementary material:**

The online version of this article (10.1007/s40820-019-0352-3) contains supplementary material, which is available to authorized users.

## Graphical abstract

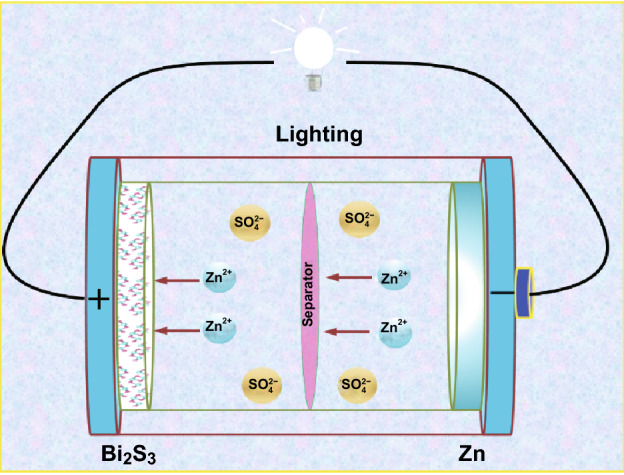

## Introduction

Energy storage devices are in great demands for the integration of renewable energy and electrical energy infrastructures due to the energy crisis and environmental pollution [1–4]. Poised as the most successful commercial energy storage devices, lithium-ion batteries (LIB) are widely used due to their rechargeability and high energy density [5–7]. However, as the usage of LIB increases, there are growing concerns regarding the safety issues of flammable organic electrolytes and the availability of lithium resources [8, 9]. To reduce such heavy reliance on LIB, there is a renewed interest in alternative energy storage devices, especially those that utilize aqueous electrolyte, i.e., aqueous rechargeable battery (ARB) [10–13]. Among these ARBs, zinc ion batteries (ZIBs) are particularly attractive as zinc displays water compatibility, natural abundance, relatively low redox potential (− 0.76 V vs. SHE) and high theoretical capacity (820 mAh g^−1^) [14–16]. Despite these numerous advantages, cathode material selection is highly stringent which poses significant difficulties in developing advanced cathode materials that show both high energy density and long cycling stability.

The most widely studied ZIBs cathode materials remain to be metal oxides such as vanadium-based oxides and manganese-based oxides [17–21]. Despite many successful demonstrations of vanadium-based and manganese-based oxides in ZIBs application, one main concern for these materials is the presence of high negative charge density O^2−^ from M–O where M = Mn or V. Such concern is amplified for ZIBs as compared to LIB due to the shuttling of densely positive charged Zn^2+^ across the material which may lead to strong electrostatic forces between O^2−^ and Zn^2+^ [16, 22]. The strongly electrostatically “glued” Zn would then be unable to be fully removed during the charging process which could eventually lead to two major issues; (1) high initial irreversible loss, and (2) poor cyclic stability. While such an issue can be alleviated with the incorporation of structural water in the interlayer spacing as an electrostatic shield, alternative strategies remain scarce [23, 24]. An alternative strategy to minimize the impact of electrostatic “adhesion” of Zn^2+^ onto O^2−^ is to replace oxide in metal oxide with sulfide. Even though metal sulfides are less studied in ZIBs than metal oxide, the anion replacement from O^2−^ to S^2−^ may reduce the tendency of these electrostatic “adhesion” and therefore leading to improved cyclic stability and minimizing the initial irreversible capacity loss [16, 22]. Furthermore, the lack in metal sulfide studies provides excellent explorative opportunities which could potentially lead toward the development of high-performing ZIBs cathode with high cyclic stability. While there are a few reports on metal sulfides for ZIBs application such as VS_2_ [25], and this small material repertoire requires urgent expansion and further exploration. Bi_2_S_3_ is a semiconductor material with a narrow band gap of 1.3 eV, and high ionic conductivity which have attracted significant attention in electrochemistry application. In particular, the highly anisotropic Bi_4_S_6_ layers that are held together by weak van der Waals interaction provides sufficient pathway for foreign ions to intercalate into. As such, it has been studied as lithium-ion battery, and sodium ion battery cathodes [26, 27]. Such layered structure presents exciting opportunity for ion intercalation which may provide possible Zn^2+^ storage.

Hence, motivated by this phenomenon, Bi_2_S_3_ is investigated as ZIBs cathode in this work. Bi_2_S_3_ nanoparticles were synthesized using a facile chemical method followed by calcining. Bi_2_S_3_ with layered structure offers paths for fast diffusion and occupancy of Zn^2+^, and also shows good cyclic stability. The as-prepared Bi_2_S_3_ delivers a high capacity of 161 mAh g^−1^ at a current density of 0.2 A g^−1^ and exhibits enhanced cyclic stability (100% retention after 100 cycles at 0.2 A g^−1^) as the cathode for ZIBs. The energy storage mechanism of the Bi_2_S_3_ electrode is revealed by a series of measurements. Results demonstrate that the capacitive process and the intercalation/deintercalation of Zn^2+^ in the Bi_2_S_3_ interlayer occur during discharging/charging processes. Our finding shows that Bi_2_S_3_ is a promising cathode material with high capacity and good stability for the development of high-performance aqueous Zn ion battery system.

## Experimental Section

### Chemicals

Na_2_S, Bi(NO_3_)_3_·5H_2_O, and ZnSO_4_·7H_2_O were purchased from Sigma-Aldrich. Acetic acid was purchased from Fluka. Carbon paper (0.18 mm, 77% porosity) was purchased from Ce-Tech Co. Ltd.

### Synthesis of Bi_2_S_3_ Nanoparticles

Bi_2_S_3_ nanoparticles were synthesized by a simple chemical reaction followed by a simple calcining method. Typically, 0.97 g of Bi(NO_3_)_3_·5H_2_O was dissolved into 100 mL H_2_O containing 9 mL acetic acid. Then, 30 mL of Na_2_S solution (0.003 mol) was added into the above solution and kept for stirring 2 h. The solid were collected by centrifugation, washed with ethanol and distilled water for three times, and then dried at 60 °C. The dried solid was then heat-treated in a vacuum oven at 200 °C for 3 h to yield crystallized Bi_2_S_3_ nanoparticles.

### Characterization

The powder X-ray diffraction (XRD) pattern was measured by a powder diffractometer (Bruker D8 Advanced Diffractometer System) with a Cu Kα (1.5418 Å) source. Scanning electron microscopy (SEM) images were recorded on a ZEISS SEM Supra 40 (5 kV). Transmission electron microscopy (TEM) was performed on a JEOL-3010 (300 kV acceleration voltage). TEM samples were prepared by dripping the sample solutions onto a copper grid. Surface composition was studied by X-ray photoelectron spectroscopy (XPS) using a Kratos Analytical Axis UltraDLD UHV spectrometer with a monochromatized Al Ka X-ray source (1486.6 eV) scanning a spot size of 700 µm by 300 µm.

### Electrochemical Measurements

All electrochemical tests were tested using an electrochemical station (Bio-logic VMP 3) at room temperature. A CR2025-type coin cell was constructed to evaluate the electrochemical performance. The as-prepared Bi_2_S_3_ nanoparticles were mixed with carbon black and polyvinyl difluoride in a 7:2:1 weight ratio with N-methyl-2-pyrrolidone. The mixture was hand-grinded and then coated onto the carbon paper, and finally dried at 80 °C for further use as cathode. Zn foil was used as the anode and filter paper was applied as the separator. 2 M of ZnSO_4_ was employed as the electrolyte. For both cyclic voltammetry and charge/discharge tests, the voltage was measured in the range of 0.4–1.2 V. The current densities of 0.2, 0.3, 0.4, 0.5, 0.6, 0.8, 1, 2, 3, 4, 5, 6, 8, and 10 A g^−1^ were selected for charge/discharge test. Electrochemical impedance spectroscopy was tested in the frequency range from 0.01 to 10^5^ Hz. Specific capacity, energy density, and power density were determined using the mass of the active material from the cathode. The mass of the active material is about 1 mg, pasted onto a 1.2 cm in diameter carbon paper.

## Results and Discussion

**Fig. 1:**
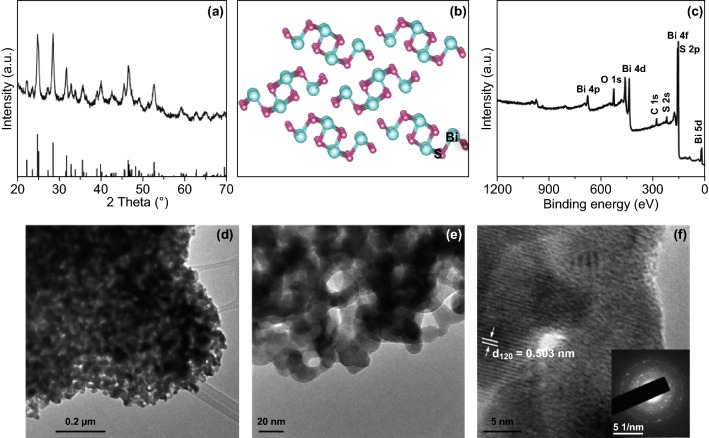
**a** XRD pattern, **b** crystallographic structure, **c** XPS spectrum, **d**, **e** TEM images, and **f** High-resolution TEM image of Bi_2_S_3_ nanoparticles (inset of the SAED image)

The Bi_2_S_3_ nanoparticles were synthesized via a simple chemical reaction followed by calcination. The XRD pattern of the obtained Bi_2_S_3_ nanoparticles displays a high degree of crystallization. All the diffraction peaks of the obtained sample can be indexed to orthorhombic Bi_2_S_3_ (JCPDS No. 17-0320) (Fig. 1a). The crystal structure of Bi_2_S_3_ is shown in Fig. 1b, which consisted of sheets of atoms parallel to the *z*-axis with each S surrounded by three Bi atoms and each Bi atom surrounded by three S atoms [28]. Also, sufficient interlayer spacing in the layers offers paths for diffusion and occupancy of foreign ions to storage energy. XPS spectrum in Fig. 1c confirms that the synthesized material is mainly composed of S and Bi elements (C and O signals come from the reference sample and absorbed oxygen).

TEM images show that the Bi_2_S_3_ exhibits nanoparticle-like morphology (Fig. 1d, e). The size of the nanoparticles is in the range of 10–50 nm, and the nanosized structure could suppress volume expansions during intercalation and deintercalation of ions [29]. The lattice distance of 0.503 nm corresponds to the (120) crystal plane of Bi_2_S_3_ in high-resolution TEM image (Fig. 1f). The SAED in the inset of Fig. 1f confirms the polycrystalline nature of the synthesized Bi_2_S_3_ nanoparticles. Further microstructural features of the synthesized Bi_2_S_3_ samples were investigated by N_2_ adsorption–desorption isotherms as shown in Fig. S1a. The type IV isotherm (IUPAC definition) is obtained with a Brunauer–Emmett–Teller surface area of 16 m^2^ g^−1^, and the H1 hysteresis loop demonstrates the presence of mesopores [30]. The pore size distribution (Fig. S1b) shows the mesopores centered at 28 nm, formed by the aggregated nanoparticles. Originating from the high crystallinity, nanosized structure and mesopores, the synthesized Bi_2_S_3_ is expected to show good electrochemical performance.

**Fig. 2:**
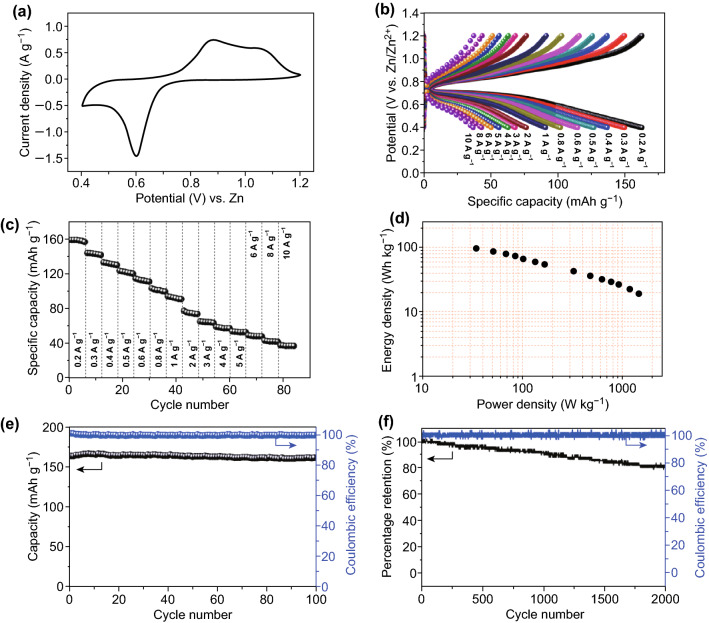
**a** CV curve at 0.5 mV s^−1^. **b** The charge/discharge curves. **c** Rate performance of Zn/Bi_2_S_3_ cell at current densities from 0.2 to 10 A g^−1^. **d** Ragone plot. **e**, **f** The long-term cycling performance at current density of 0.2 A g^−1^ and 1 A g^−1^

The electrochemical performance of the material was assessed by assembling a Zn/Bi_2_S_3_ cell using Bi_2_S_3_ as cathode and Zn foil as anode in an aqueous solution of 2 M ZnSO_4_. Figure 2a shows the cyclic voltammetry (CV) curve of Zn/Bi_2_S_3_ cell at scan rate of 0.5 mV s^−1^ in the voltage window of 0.4–1.2 V. Obvious redox peaks could be observed, which may be related to the interaction between Bi_2_S_3_ and Zn ions. In addition, galvanostatic discharge/charge (GCD) profiles are in accordance with the CV curves, which show one plateau around 0.6 V (Fig. 2b). Zn/Bi_2_S_3_ cell shows a high discharge capacity of 161 mA h g^−1^ at 0.2 A g^−1^. It also shows excellent rate performance, demonstrating high capacities of 143, 132, 121, 113, and 101 mAh g^−1^ at 0.3, 0.4, 0.5, 0.6, and 0.8 A g^−1^, respectively (Fig. 2c).

The energy and power densities of the Zn/Bi_2_S_3_ battery are calculated as shown in Fig. 2d. It can be seen that the assembled battery shows a maximum energy density of 105 Wh kg^−1^, and also it delivers a maximum power density of 1455 W kg^−1^. For the long-term cycling stability, the Zn/Bi_2_S_3_ cell obtains a cyclic retention of 100% up to 100 cycles at a low current density of 0.2 A g^−1^, with the corresponding columbic efficiency approaching 100% (Fig. 2e). The capacity remains 80.3% after 2000 cycles at current density of 1 A g^−1^ (Fig. 2f). Also, we tested the XRD of the cycled Bi_2_S_3_, and the result shows that the sample is stable because it shows similar XRD pattern to the fresh one (Fig. S2). The good stability indicates that the Bi_2_S_3_ is a promising material for Zn ion battery application.

**Fig. 3:**
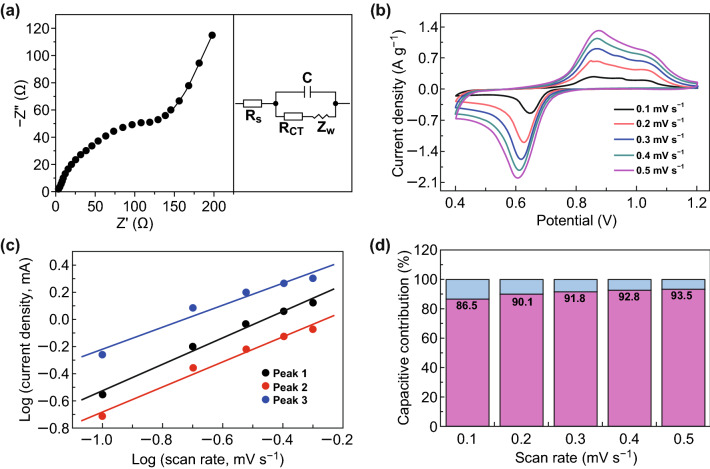
**a** Electrochemical impedance spectroscopy (EIS). **b** CV curves at different scan rates. **c** The corresponding plots of log (peak current) versus log (scan rate) at the redox peaks. **d** The calculated capacitive contributions

Electrochemical impedance spectroscopy (EIS) measurements are performed on the Zn/Bi_2_S_3_ battery to study the detailed reaction kinetics. Figure 3a exhibits the Nyquist plots with a semicircle (at high frequency) involved with charge transfer and a sloped line associated with ion diffusion (at low frequency) [31]. In the equivalent circuit, by fitting, Bi_2_S_3_ shows low resistance of *R*s (equivalent series resistance, 5.02 OhΩ), *R*_CT_ (charge transfer resistance, 4.1 OhΩ), and *Z*_W_ (936.2 OhΩ), illustrating the fast reaction kinetics. The fast reaction kinetics could be attributed to the reduced electrostatic “adhesion” of Zn^2+^ onto anion S^2−^ which leads to fast diffusion of Zn^2+^. To reveal the electrochemical kinetics of the Bi_2_S_3_ electrode, CV curves at different scan rates from 0.1 to 0.5 mV s^−1^ are studied in Fig. 3b. In each curve, three peaks were observed. An equation for analyzing the electrochemical kinetics processes, based on the peak currents (*i*) and scan rates (*v*) is shown as Eq. 1 [32, 33]:

1 $$i = \, av^{b}$$

which can be equally written as Eq. 2

2 $$\log \left( i \right) \, = \, b\log \left( v \right) \, + \, \log \left( a \right)$$

where *b* is defined as the slope of log(*i*) versus log(*v*) curve. Typically, the value of *b* (0.5–1) is related to the type of electrochemical process. When b value reaches 0.5, it indicates that the electrochemical kinetics process is dominated by ionic diffusion. Surface capacitive effects become dominant as the b value is 1. From Fig. 3c, the *b* values for peak 1, 2, and 3 are determined to be 0.81, 0.91, and 0.97, separately. These b values hint that the ionic diffusion along with surface capacitive effects would control the electrochemical kinetics reaction for Zn/Bi_2_S_3_ cell. The contribution from capacitor-like and diffusion-controlled processes can further be determined by Eq. 3 [34, 35]:

3 $$i \, = k_{1} v + k_{2} v^{1/2}$$

which can be rewritten in Eq. 4

4 $$i/v^{1/2} = k_{1} v^{1/2} + k_{2}$$

where *i* is related to the total current response, *k*_*1*_*v* represents current from surface capacitive effects, and *k*_2_*v*^1/2^ means current because of ionic diffusion process. As *k*_1_ could be achieved via fitting *i*/*v*^1/2^ versus *v*^1/2^ plots, the contribution from capacitive effect is determined to be 86.5% at scan rate of 0.1 mV s^−1^. As scan rate increases, the percentage of capacitive contribution is up to 90.1%, 91.8%, 92.8%, and 93.5% at 0.2, 0.3, 0.4, and 0.5 mV s^−1^, respectively (Fig. 3d). The results suggest that the capacitive contribution is dominant, and the capacitive contribution ratios gradually increase with an increase in scan rate.

**Fig. 4:**
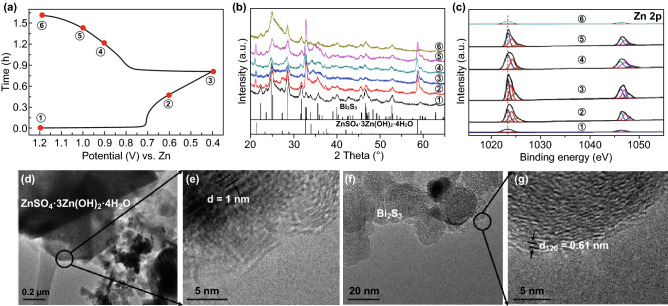
**a** Charge/discharge curve at 0.2 A g^−1^. **b** The corresponding ex situ XRD patterns. **c** XPS spectra Zn 2p at selected states. **d**, **e** TEM images of ZnSO_4_·3Zn(OH)_2_·4H_2_O. **f**, **g** TEM images of Bi_2_S_3_ at the fully discharged state

The storage mechanism is investigated by ex situ XRD, XPS spectra, SEM–EDX elemental mappings and TEM. Before charge/discharge process, we performed CV for 30 cycles to get a stable state. From the charge/discharge processes as shown in Fig. 4a, b, it indicates that ZnSO_4_·3Zn(OH)_2_·4H_2_O (JCPDS No. 09-0204) is successively formed during the discharge process. Subsequently, ZnSO_4_·3Zn(OH)_2_·4H_2_O disappears after being fully charged to 1.2 V. These results demonstrate the reversible formation/decomposition of ZnSO_4_·3Zn(OH)_2_·4H_2_O during the discharge/charge process. The storage mechanism was also investigated by the XPS spectra at different charge/discharge states (Fig. 4c). It should be noted that a small amount of Zn^2+^ ions in Bi_2_S_3_ is detected after 30 cycles, suggesting that some of Zn^2+^ ions were trapped into Bi_2_S_3_ (state ① in Fig. 4c). During the discharging process, two pairs of Zn^2+^ peaks gradually appear at 1024.1/1047.1 eV and 1024.8/1047.9 eV, which are attributed to the Zn^2+^ in Zn(OH)_2_ and ZnSO_4_ from ZnSO_4_·3Zn(OH)_2_·4H_2_O, separately [19]. Meantime, the intensity about the Zn^2+^ peaks at 1023.3/1046.3 eV associated with the inserted Zn^2+^ in Bi_2_S_3_ increases, confirming the Zn^2+^ insertion process.

During the charging process, the peaks associated with the Zn^2+^ in Zn(OH)_2_ and ZnSO_4_ from ZnSO_4_·3Zn(OH)_2_·4H_2_O gradually disappear, further confirming the reversible conversion of ZnSO_4_·3Zn(OH)_2_·4H_2_O, consistent with the XRD result. Simultaneously, the peak intensity gradually decreases for the inserted Zn^2+^ in Bi_2_S_3_. It indicates the continuous and reversible intercalation/extraction of Zn^2+^ in Bi_2_S_3_ during the electrochemical processes. SEM–EDX elemental mappings of the fully discharged Bi_2_S_3_ electrode are shown in Fig. S3. Obviously, elemental Zn is uniformly distributed in the Bi_2_S_3_ nanoparticles, which confirms the insertion mechanism of Zn ion into Bi_2_S_3_ layers.

The structural evolution of Bi_2_S_3_ electrode was further investigated by TEM analysis. For the Bi_2_S_3_ electrode at the fully discharged state, in Fig. 4d, e, nanoplates could be observed, and the observed lattice fringes with interplanar distances of ca. 1 nm correspond to the plane of ZnSO_4_·3Zn(OH)_2_·4H_2_O, indicating the generation of ZnSO_4_·3Zn(OH)_2_·4H_2_O during the discharge process, consistent with the XRD and XPS analysis. Also, Bi_2_S_3_ with nanoparticles structure could be observed. The lattice spacing of 0.61 nm, which was enlarged when compared with that of the fresh Bi_2_S_3_ with lattice distance of 0.503 nm, was clearly observed in the HRTEM image (Fig. 4f, g). The enlarged lattice could be attributed to the insertion of Zn^2+^. The layered structure of Bi_2_S_3_ with the weak van der Waals interaction between layers offers paths for diffusion and occupancy of Zn^2+^.

Hence, based on the collective results, the electrochemical mechanism of Zn/Bi_2_S_3_ is related to the capacitive process and the intercalation/extraction of Zn^2+^ into the Bi_2_S_3_ framework during discharging/charging process, which is summarized as follows: During the discharge process, ZnSO_4_·3Zn(OH)_2_·4H_2_O is successively formed which is confirmed by the XRD, XPS (the detected Zn^2+^ peaks in Zn(OH)_2_ and ZnSO_4_ from ZnSO_4_·3Zn(OH)_2_·4H_2_O) and TEM (the observed interplanar distances of ca. 1 nm for ZnSO_4_·3Zn(OH)_2_·4H_2_O) results. Meantime, Zn^2+^ is inserted into Bi_2_S_3_ as revealed by the XPS (enhanced Zn^2+^ peaks intensity of the inserted Zn^2+^ in Bi_2_S_3_), TEM (enlarged lattice distance of 0.61 nm for Bi_2_S_3_), and SEM–EDX elemental mappings (uniform distribution of Zn^2+^ in the discharged Bi_2_S_3_) results. During the charging process, ZnSO_4_·3Zn(OH)_2_·4H_2_O gradually disappears. These results demonstrate the reversible formation/decomposition of ZnSO_4_·3Zn(OH)_2_·4H_2_O during the discharge/charge process. At the same time, Zn^2+^ ions are extracted from Bi_2_S_3_ as demonstrated by the decreased XPS intensity of Zn^2+^ ions. Overall, the designed Bi_2_S_3_ electrode shows high zinc ion storage performance with faster reaction kinetics, higher capacity and better long-term cycles, presenting a potentially safe, durable, and low-cost device for large-scale energy storage.

## Conclusions

In conclusion, we report a simple chemical method followed by calcining to synthesize Bi_2_S_3_ nanoparticles as cathodes for aqueous rechargeable ZIBs. The obtained Bi_2_S_3_ nanoparticles display high capacity of 161 mAh g^−1^ at current density of 0.2 A g^−1^. Good rate behavior is demonstrated, and the Zn/Bi_2_S_3_ cells show good cycling stability over 100 cycles. At high current density of 1 A g^−1^, the cell still keeps 80.3% retention after 2000 cycles. Mechanistic details of the Zn storage based on surface capacitive effects and Zn^2+^ ions insertion are demonstrated. The high capacity, good stability, and low cost make our battery promising for stationary energy storage applications.

## Electronic supplementary material

Below is the link to the electronic supplementary material.

Supplementary material 1 (PDF 299 kb)
