# Supplementary material for: Bi2S3 for Aqueous Zn Ion Battery with Enhanced Cycle Stability
Source: Nanomicro Lett. 2019 Dec 19;12:8. doi: 10.1007/s40820-019-0352-3 (PMC7770836; doi:10.1007/s40820-019-0352-3)
Supplement: Supplementary file 1 — Supplementary material 1 (PDF 299 kb) [file 40820_2019_352_MOESM1_ESM.pdf]

Supporting Information for

Bi_2S_3 for Aqueous Zn Ion Battery with Enhanced Cycle Stability

Ting Xiong [1, 2], Yinming Wang[1], Bosi Yin[1, 3], Wen Shi[1], Wee Siang Vincent Lee[1, *], Junmin Xue[1, *]

[1]National University of Singapore, Department of Materials Science and Engineering, Singapore 117573, Singapore

[2]Centre for Advanced 2D Materials and Graphene Research Centre, National University of Singapore, Singapore 117546, Singapore

[3]MIIT Key Laboratory of Critical Materials Technology for New Energy Conversion and Storage, School of Chemistry and Chemical Engineering, Harbin Institute of Technology, No. 92 West-Da Zhi Street, Harbin 150001, People's Republic of China

*Corresponding authors. E-mail: mseleew@nus.edu.sg (Wee Siang Vincent Lee); msexuejm@nus.edu.sg (Junmin Xue)

Supplementary Figures

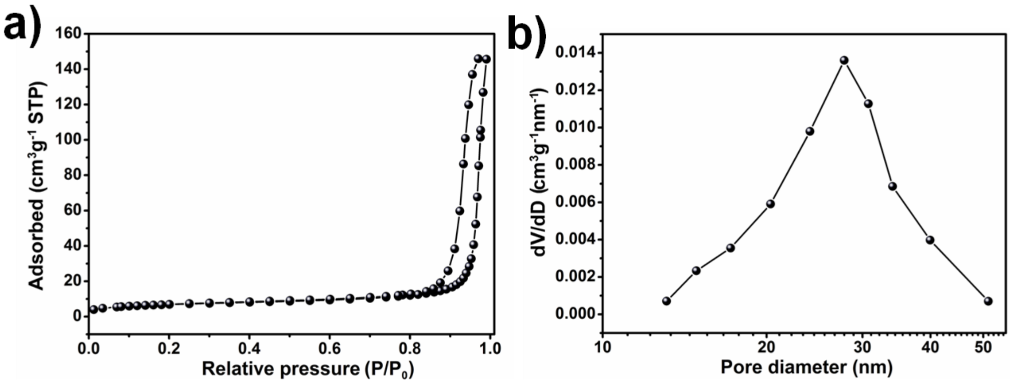

Fig. S1 a Nitrogen adsorption–desorption isotherm. **b** The corresponding pore size distribution plot

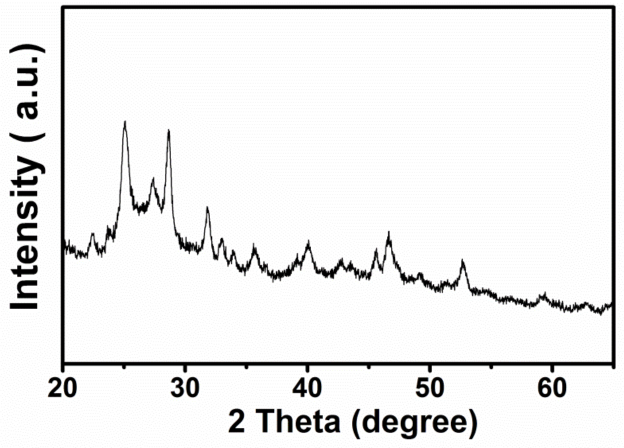

Fig. S2 XRD pattern of the cycled Bi_2S_3

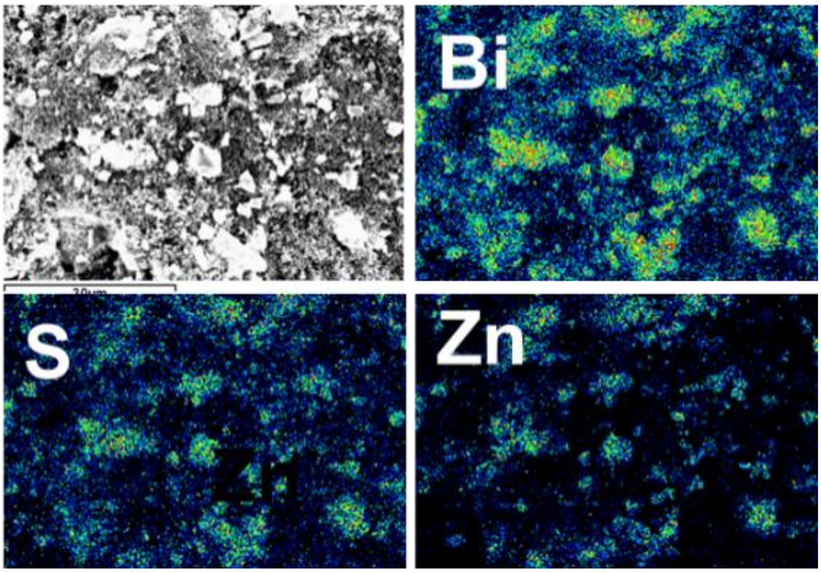

Fig. S3 SEM-EDX elemental mappings of the Bi_2S_3 electrode at the fully discharged state
